# Clearing the Air: Smoke-Free Housing Policies, Smoking, and Secondhand Smoke Exposure Among Affordable Housing Residents in Minnesota, 2014–2015

**DOI:** 10.5888/pcd13.160195

**Published:** 2016-08-18

**Authors:** John H. Kingsbury, Dawn Reckinger

**Affiliations:** Author Affiliation: Dawn Reckinger, Minnesota Department of Health, St. Paul, Minnesota.

## Abstract

**Introduction:**

During the past 30 years, local and state tobacco use control laws in the United States have helped reduce smoking prevalence and exposure to secondhand smoke, but progress among low socioeconomic populations has been slow. Implementing smoke-free housing policies in affordable housing may help address this issue. The purpose of our study was to assess how such policies affect smoking rates and exposure to secondhand smoke among residents of affordable housing.

**Methods:**

We conducted a pretest–posttest longitudinal study of 180 residents from 8 affordable housing properties in Minnesota. Participating properties agreed to adopt a smoke-free housing policy covering indoor grounds, and 3 of these properties also prohibited smoking on all outdoor grounds. Policies were implemented with assistance from local public health departments and the Statewide Health Improvement Program. Participants completed surveys one month before policy implementation and 6 months postimplementation. Surveys assessed smoking, quit attempts, and indoor and outdoor secondhand smoke exposure.

**Results:**

Results indicated a significant reduction in nonsmokers’ indoor exposure to secondhand smoke (*F*_1,144 _= 22.69, *P* < .001) and no change in outdoor exposure to secondhand smoke from Time 1 (pretest) to Time 2 (posttest) (*F*_1,140_ = 2.17, *P* = .14). However, when examining sites that only prohibited smoking indoors, we observed an increase in outdoor secondhand smoke exposure that approached significance (*F*_1,118_ = 3.76, *P* = .055). Results showed no change in quit attempts over time, but 77% of residents who smoked at pretest reported reducing the amount that they smoked at posttest, and an additional 5% reported that they had quit.

**Conclusions:**

Smoke-free housing policies may be an effective strategy to reduce exposure to indoor secondhand exposure and promote decreased cigarette smoking among residents of affordable housing.

## Introduction

Smoking remains the leading cause of preventable death in the United States, contributing to more than 480,000 deaths per year among smokers and 50,000 deaths among nonsmokers (1). During the past 30 years local and state tobacco use control laws such as those that prohibit smoking in restaurants and bars have helped reduce smoking prevalence by 40% and exposure of nonsmokers to secondhand smoke by 71% (2–5). Despite declines in these indicators for the general population, progress has been considerably slower for populations of low socioeconomic status, who experience a disproportionate burden of harm from smoking (5–7). This is particularly true in Minnesota where the rate of smoking is 121% higher among those without a high school degree than among those with more than a high school degree (8).

Because smoking rates are disproportionately high among populations of low socioeconomic status, the risk of secondhand smoke exposure among these groups is high. Results from a nationally representative study showed that nonsmokers with an annual household income of less than $20,000 were 36% more likely to have elevated serum cotinine levels — a marker of high secondhand smoke exposure — than those with an annual household income of $20,000 or more (9). Researchers have posited that a primary contributor to socioeconomic disparities in secondhand smoke exposure is unequal access to quality housing, and a primary component of quality housing is a smoke-free environment. People of low socioeconomic status have less access to smoke-free housing than do those of high socioeconomic status and consequently are more likely to live in multi-unit buildings where smoking is permitted in individual housing units (10). Consequently, people of low socioeconomic status are at increased risk of secondhand smoke exposure in the home environment, which is where people spend 69% of their time (11).

In 2007, Minnesota passed the Clean Indoor Air Act, which prohibits smoking in most indoor public spaces, including common areas in rental apartment buildings. However, this law did not prohibit smoking in individual units, leaving open the possibility that smoke from one unit could transfer to other units. Investigations of secondhand smoke in multi-unit residential buildings found nicotine concentrations in units without smokers to be comparable to those of units with smokers, suggesting that secondhand smoke infiltration from neighboring units may be substantial (12). Consistent with these findings, studies found that indoor secondhand smoke exposure among nonsmokers in such housing is common, with 53% of residents reporting any exposure (13) and 41% reporting frequent exposure (14).

Smoke-free policies for multi-unit housing can address secondhand smoke transfer by prohibiting smoking in all indoor areas, and — with a stronger policy — all outdoor areas too. Few studies have investigated the effects of smoke-free policies in multi-unit housing on secondhand smoke exposure and quit attempts, but evaluations of other smoke-free indoor policies are promising. For example, smoke-free workplace policies are effective at reducing exposure to secondhand smoke (15,16). These policies also appear to decrease cigarette smoking and promote smoking cessation. One study found that smokers who worked in environments that implemented a smoke-free workplace policy were nearly twice as likely to quit as workers without a smoke-free workplace policy, and those who continued smoking reported a significant reduction in number of cigarettes smoked daily (17).

Findings from these studies suggest that policies prohibiting smoking in multi-unit affordable housing could help decrease tobacco use and secondhand smoke exposure among residents and consequently reduce socioeconomic tobacco-related health disparities (18). However, little is known about the effects of smoke-free housing policies on smoking and secondhand smoke exposure among low-income populations. To our knowledge, only 2 studies have examined this issue with a focus on low-income residents, and these studies were limited by the use of retrospective self-reports of pre-policy smoking (14) and a policy that included indefinite grandfathering (ie, allowing smokers residing in the apartment at the time the policy was implemented to continue smoking in their units) (19). A recent review noted the shortage of research on smoking and secondhand smoke exposure in affordable housing and called for more prospective studies on the effect of smoke-free housing policies on residents’ smoking behavior (20). The purpose of our study was to determine the effects of smoke-free policies in multi-unit housing on secondhand smoke exposure and tobacco use among low-income populations.

## Methods

Eight public housing properties participated in a longitudinal pretest–posttest evaluation. The properties were from 4 regions of Minnesota and comprised a mix of urban and rural communities (population range: 1,700–86,000). A large proportion of residents at participating properties were seniors. The smoke-free policies implemented at each property prohibited smoking in all indoor areas; 3 of the properties also prohibited smoking on all outdoor grounds. Residents were notified of the smoke-free policy between 3 and 12 months prior to implementation. Policies were implemented with the assistance of the staff of the local public health department and with the support of the Statewide Health Improvement Program, a grant program that focuses on increasing physical activity and healthy eating, and reducing tobacco use and exposure to secondhand smoke in Minnesota. Sites were recruited with the help of staff members of the local public health department, who helped identify properties that had agreed to implement a smoke-free multi-unit housing policy. Residents were recruited via flyers posted on bulletin boards at each site. The Minnesota Department of Health institutional review board approved the study.

Time 1 (pretest) data were collected at each site one month before implementation of the smoke-free multi-unit housing policy (from February 2014 through March 2015), and Time 2 (posttest) data were collected 6 months postimplementation (from September 2014 through October 2015). Time 1 surveys were distributed door to door to all residents in participating buildings. Time 2 surveys were distributed only to Time 1 respondents who still lived at the site. Sites could choose from 2 compensation options for participants. Five sites chose to have participants receive a $15 gift card for completing the Time 1 survey and a $20 gift card for completing the Time 2 survey. The other 3 sites chose to do random drawings for several $50 gift cards at both Time 1 and Time 2. Smoking cessation resources were offered to all resident smokers before policy implementation.

A total of 578 residents received a Time 1 survey, and 289 residents completed it (50% response rate). Of these 289 residents, 25 moved out before the Time 2 survey. Of the remaining 264 Time 1 respondents, 180 (62.3%) also completed a Time 2 survey, yielding a 68.2% response rate at Time 2 and a 62.3% retention rate.

The Time 1 survey assessed secondhand smoke exposure, cigarette smoking, quit attempts, and participant demographics. The Time 2 survey was identical to the Time 1 survey, except for additional items assessing smoking cessation methods and reasons for reducing smoking or trying to quit. Demographic characteristics collected were age, race/ethnicity, highest level of education (1 = less than high school degree, 2 = high school graduate or general equivalency degree, 3 = some college, associate degree or vocational, technical, or business school, 4 = bachelor degree or higher, 5 = PhD, MD, JD, or other professional degree), and annual household income (1 = ≤$10,000, 2 = $10,001-$20,000, 3 = $20,001-$25,000, 4 = $25,001-$35,000, 5 = $35,001-$50,000, 6 = $50,001-$75,000, 7 = ≥$75,000). Questions related to secondhand smoke assessed both indoor and outdoor exposure. For indoor exposure, participants were asked 1) how often they smelled or breathed secondhand smoke when inside their own apartments and 2) how often they smelled or breathed secondhand smoke in shared areas such as hallways, stairwells, community rooms, or laundry rooms. For outdoor exposure, they were asked how often they smelled or breathed secondhand smoke in parking lots, lawns, or playgrounds. Responses for indoor and outdoor exposure were rated on a scale of 1 to 5 (1 = never, 2 = hardly ever, 3 = a few times a month, 4 = a few times a week, 5 = every day). Responses to the 2 indoor exposure questions were aggregated to form an indoor exposure scale (α = .74). Smoking status was assessed by asking if residents had smoked 100 cigarettes in their lifetime (yes or no) and if they currently smoked cigarettes (responses: every day, some days, not at all). Participants were classified as current smokers if they reported smoking 100 cigarettes during their lifetime and currently smoked cigarettes “some days” or “every day.” Residents’ quit attempts were assessed by asking whether in the past 6 months they had stopped smoking for one day or longer because they were trying to quit (yes or no). In the Time 2 survey, participants were asked if the amount they smoked changed in the past 6 months (responses: smoked more, smoked less, smoked about the same, quit smoking). Smokers who had reduced their smoking or had tried to quit in the past 6 months were asked their primary reasons for doing so (responses were family reasons, health, cost, or inconvenience [ie, not allowed to smoke in their apartments]; residents could choose more than one response).

Data were analyzed with SPSS version 22.0 (Stata Corp LP); significance was set at *P* < .05. Independent sample *t* tests were used to identify differences in demographics between residents lost to follow-up between the Time 1 and Time 2 surveys and those who were retained. Time 1 to Time 2 changes in nonsmokers’ reported indoor and outdoor exposure to secondhand smoke were examined by using repeated-measures ANOVAs. These analyses were followed by another repeated-measures ANOVA to test for differences in secondhand smoke exposure outdoors among sites that did not prohibit smoking outdoors. Differences in quit attempts from Time 1 to Time 2 were tested using McNemar’s test.

## Results

Demographic characteristics of participants retained at Time 2 were not significantly different from those lost to follow-up, except for race/ethnicity, education, and income (Table). Among those retained at Time 2, race, education, and income were not significantly related to the outcomes of interest (all *P *> .05).

**Table:** Demographic Characteristics of Participants (N = 289), Study of Secondhand Smoke Exposure Among Affordable Housing Residents, Minnesota, 2014–2015 **a**

Characteristic	Completed Time 1 and Time 2 Surveys, n = 180	Completed Time 1 Survey Only, n = 109
**Sex**		
Male	58 (32.2)	27 (24.8)
Female	122 (67.8)	82 (75.2)
**Race^b^^,^ c ^,^ d**		
White	130 (72.6)	90 (87.4)
Black	42 (23.5)	8 (7.8)
Other	7 (4.0)	5 (4.8)
**Mean age, y**	62.9	62.4
**Annual income, $^d^^,^ e**		
<10,000	62 (38.8)	20 (20.8)
10,000–20,000	54 (33.8)	31 (32.3)
>20,000	44 (27.5)	45 (46.9)
**Education^d^**		
<High school diploma	51 (29.3)	15 (14.2)
High school or general equivalency degree	49 (28.2)	20 (18.9)
Some college or associate’s degree	43 (24.7)	35 (33.0)
≥Bachelor’s degree^f^	31 (17.8)	36 (34.0)
**Smoking status**		
Nonsmoker	153 (85.0)	87 (79.8)
Smoker	27 (15.0)	22 (20.2)

a Values are expressed as no. (%), unless otherwise indicated; values for n may not sum to totals because of missing values.

b Independent samples *t* tests were used to test significant differences between the final sample and participants who were lost to follow-up.

c Race categories other than white and black (ie, Asian or Asian American, American Indian or Alaskan Native, Native Hawaiian or Pacific Islander, other) were combined because of small numbers.

d *P* < .001.

e Income categories greater than $20,000 were collapsed into one category due to the small number of participants reporting higher levels of income.

f Bachelor’s degree or higher education includes those who reported a bachelor’s degree and a PhD or other professional degree as their highest level of education.

The final sample of 180 was 68% female, 73% white, 23% black, and had a mean age of 63 years (range: 21–99). The proportion of current smokers at Time 1 was 15%. Twenty-nine percent of the sample had less than a high school education, 82% had less than a bachelor’s degree, and 72.5% earned $20,000 a year or less.

A repeated-measures ANOVA indicated a significant decrease in nonsmokers’ reported exposure to secondhand smoke indoors from Time 1 (44.0%) to Time 2 (23.6%), *F*_1,144_ = 22.69, *P* < .001 (Figure). Conversely, there was no significant difference from Time 1 to Time 2 in outdoor secondhand smoke exposure, *F*(_1,140_) = 2.17, *P* = .14. Follow-up analyses tested for a difference in outdoor secondhand smoke exposure among nonsmokers living in properties that did not prohibit smoking outdoors (n = 119). Results revealed a marginally significant increase in outdoor secondhand smoke exposure from Time 1 to Time 2, *F*(_1,118_) = 3.76, *P* = .055 (Figure).

**Figure:**
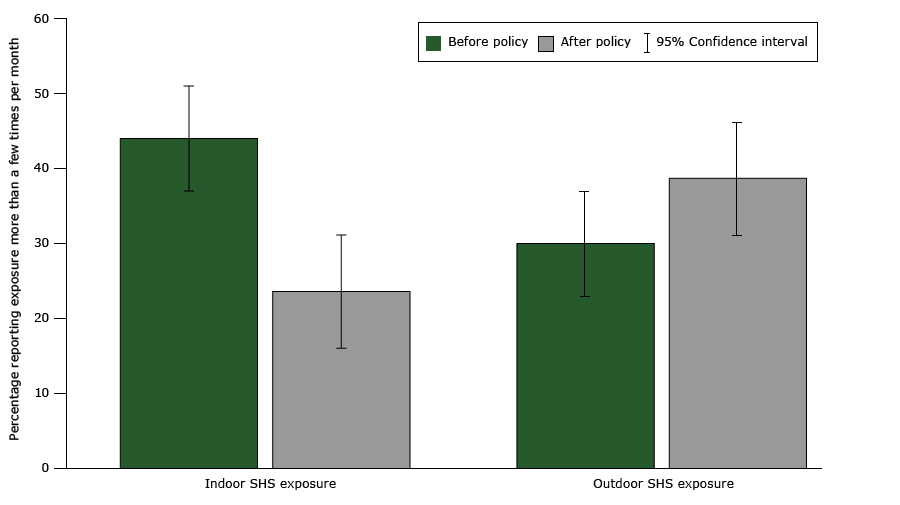
Nonsmokers' indoor and outdoor exposure to secondhand smoke. Indoor secondhand smoke exposure comparison is between all nonsmokers (N = 145), at Time 1 and Time 2, Minnesota, 2014–2015. Outdoor secondhand smoke exposure comparison is between nonsmokers from sites that did not prohibit smoking on all grounds (N = 119), at Time 1 and Time 2. Error bars indicate 95% confidence intervals (CIs). Abbreviation: SHS, secondhand smoke. **Table:** 

Type of Exposure	Nonsmokers Who Reported Secondhand Smoke Exposure More Than a Few Times Per Month, % (95% Confidence Interval)	
	Before Policy	After Policy
Indoor	44.0 (35.8–52.2)	23.6 (16.5–30.7)
Outdoor	30.0 (21.8–38.2)	38.7 (30.5–47.5)

Results from McNemar’s test indicated no significant difference in the proportion of smokers who reported making a quit attempt in the past 6 months at Time 1 (77.8%) and Time 2 (63.0%), *P* > .05.

At Time 2, 77% of smokers reported reducing the amount that they smoked in the past 6 months, and an additional 5% reported that they had quit. Those who had quit, tried to quit, or reduced the amount they had smoked in the past 6 months reported their reasons for doing so at Time 2. The most commonly cited reasons for quitting, trying to quit, or reducing smoking were inconvenience attributable to not being able to smoke in the apartment (52%) and health (52%). The next most common reasons were cost (40%) and family (25%).

## Discussion

Results of this study suggest that smoke-free policies in affordable multi-unit housing can reduce residents’ exposure to secondhand indoor smoke. This finding is consistent with that of Pizacani and colleagues (14), who found that frequent secondhand smoke exposure inside apartments dropped from 41% to 17% after implementation of a smoke-free policy. Although indoor exposure to secondhand smoke decreased significantly from Time 1 to Time 2, 23.6% of residents reported secondhand smoke exposure at least a few times per month after the policy, suggesting enforcement and compliance issues. Lack of compliance with the policy can undermine health benefits associated with smoke-free housing. Future studies should use long-term follow-up surveys to examine whether compliance issues diminish over time and also conduct landlord interviews to investigate resident complaints and enforcement practices.

Analyses examining changes in outdoor exposure to secondhand smoke showed no difference from Time 1 to Time 2 for all nonsmokers. However, further analyses indicated a marginal increase in outdoor secondhand smoke exposure among nonsmokers living in properties that did not restrict smoking outdoors. These findings suggest that secondhand smoke exposure may increase in locations not covered by the smoke-free policy after its implementation. The observed increase in outdoor secondhand smoke exposure may be due to smokers obeying the smoke-free policy but smoking in locations that are unavoidable by nonsmoking residents (eg, outside the front entryway, in the parking lot). These findings highlight the importance of implementing comprehensive smoke-free policies that cover all property grounds rather than indoors only.

There was no change in quit attempts in the past 6 months from Time 1 to Time 2. Residents were informed well in advance of the implementation date — 6 months or more, in most cases — that the smoke-free policy would be implemented. Therefore, residents may have attempted to quit smoking before the implementation date in anticipation of the policy going into effect, resulting in an inflated number of baseline quit attempts. The proportion of smokers who made a quit attempt in the 6 months before implementation (77.8%) was substantially greater than the proportion of smokers in the general population who are seniors (mean age was 63 in the current study) and made a quit attempt in the past 12 months (38.8%) (1). Future studies should assess quit attempts before residents are informed about a forthcoming smoke-free policy to establish baseline quit attempts.

The percentage of Time 1 smokers who successfully quit smoking at Time 2 (4.5%) was comparable to previous estimates of the annual quit rate for smokers in the general population in the absence of an intervention (2.6%) (14), suggesting that the policy did not substantially increase quitting. These findings are inconsistent with a recent evaluation of smoke-free multi-unit housing policies among low-income residents that found a quit rate of 22.1% post implementation; however, this quit rate was observed 18 months following implementation of the smoke-free policy, a follow-up period 3 times as long as in this study (14). Although quitting smoking did not increase in our study, most smokers (77%) did report reducing the amount that they smoked at the 6-month follow up, a figure that is substantially greater than that found in the Pizacani et al study (49%) (14). This finding suggests that, although residents were not more apt to make a quit attempt or successfully quit smoking, they did reduce their smoking. Residents may have planned to reduce the amount they smoked before making a quit attempt with the hope that they would increase their chances of success. Previous research has found that, although the health benefits of reducing smoking are modest to negligible (21–23), reducing smoking is predictive of later cessation among smokers who are motivated to quit (24). Thus, it is conceivable that many smoking residents were in the early stages of quitting at the Time 2 assessment. Long-term follow-ups with multiple assessments are needed to test this hypothesis.

Among Time 1 smokers who had quit, tried to quit, or reduced the amount that they smoked at Time 2, the most commonly cited reason for changing their smoking behavior was the inconvenience of not being allowed to smoke in their apartment. Having to go outside or off property grounds to smoke — a direct result of a smoke-free housing policy — was enough of a deterrent that residents reported smoking less after the policy was implemented. This finding has implications for cessation interventions, because inconvenience was cited as frequently as health, and more so than other frequently targeted cessation motivations, such as familial concerns and the high cost of smoking.

A limitation of this study was that we did not measure the number of cigarettes smoked at Time 1 and Time 2. Results suggested a reduction in smoking from Time 1 to Time 2, but it is possible that residents knew the study was focused on smoking behavior and responded in a socially desirable way — by reporting that they were smoking less than they actually were after the policy. Future studies should include objective measures of smoking (eg, blood cotinine) and smoking reduction measures that assess reduction by 50% or more to facilitate comparison with other smoking interventions (25,26). Second, we had no control group in this study, which limits inferences about causality. For example, a substantial proportion of smokers reported reduced smoking at Time 2, but the lack of a control group made it difficult to determine whether this effect was significant. Post hoc 1-sample *t* tests were conducted to test whether the proportion of smokers who quit or reduced their smoking at Time 2 (82%) was significantly different from the proportion of smokers in the general population who made a quit attempt in the past year (ie, stopped smoking for 1 day or longer when trying to quit [38.8%]) (1). Results of this test did indicate a significant difference (*P* < .001); however, larger studies that include a valid comparison group (eg, affordable housing residents living in buildings that have not implemented a smoke-free policy) are needed to corroborate these findings. Third, a convenience sample composed primarily of senior residents was used; therefore, results may not generalize to other affordable housing populations. Fourth, this study did not assess whether smokers changed where they smoked post implementation. These data would have provided more insight into the observed effects on indoor and outdoor secondhand smoke exposure and should be collected in future studies.

Populations of low socioeconomic status have disproportionately high rates of smoking and exposure to secondhand smoke (3,5,6). Results from this study suggest that smoke-free multi-unit housing policies in affordable housing properties could reduce secondhand smoke exposure and cigarette smoking among low-income populations. Although more research is needed to investigate strategies to address compliance and enforcement issues, implementing smoke-free multi-unit housing policies in affordable housing may be a promising step toward eliminating tobacco-related disparities.
